# Economic effects of livestock disease burden in Ethiopia: A computable general equilibrium analysis

**DOI:** 10.1371/journal.pone.0310268

**Published:** 2024-12-31

**Authors:** Amanda M. Countryman, Taís C. de Menezes, Dustin L. Pendell, Jonathan Rushton, Thomas L. Marsh

**Affiliations:** 1 Department of Agricultural and Resource Economics, Colorado State University, Fort Collins, Colorado, United States of America; 2 Department of Agricultural Economics, Kansas State University, Manhattan, Kansas, United States of America; 3 Global Burden of Animal Diseases, Livestock and One Health Department, University of Liverpool, Liverpool, Merseyside, England; 4 School of Economic Sciences and Paul G. Allen School for Global Health, Washington State University, Pullman, Washington, United States of America; Federal University of Technology Minna, NIGERIA

## Abstract

The burden of animal disease is widespread globally and is especially severe for developing countries dependent on livestock production. Ethiopia has the largest livestock population in Africa and the second-largest human population on the continent. Ethiopia is one of the fastest-growing economies in Africa; however, much of the population still lives in extreme poverty, and most households depend on agriculture. Animal disease negatively affects domestic livestock production and limits growth potential across the domestic agricultural supply chain. This research investigates the economic effects of livestock disease burden in Ethiopia by employing a computable general equilibrium model in tandem with animal health loss estimates from a compartmental livestock population model. Two scenarios for disease burden are simulated to understand the effects of improved animal health on domestic production, prices, trade, gross domestic product (GDP), and economic welfare in Ethiopia. Results show that improved animal health may increase Ethiopian GDP by up to 3.6%, which improves national welfare by approximately $US 2.5 billion. This research illustrates the economic effects of improved livestock health, which is critical for Ethiopian households and the national economy.

## Introduction

Animal diseases impose risks and burdens on food safety, food security, and economic activity. The investigation of the economic impacts caused by animal diseases is complex because each disease has its own distinguishing features, affecting different species and spreading in distinct ways. Some important animal diseases that mostly affect the livestock sector are paratuberculosis, bovine tuberculosis, and brucellosis in bovines; scrapie in sheep and goats; avian influenza, Newcastle disease, and Marek’s disease in chickens; and foot-and-mouth disease (FMD) in cloven-hoofed animals [1]. Combined, animal diseases across species negatively affect production and impose costs across the livestock and animal product supply chain [2, 3]. Developing countries are particularly vulnerable to the negative consequences of animal disease [4, 5]. Ethiopia maintains the largest livestock population in Africa and is an important livestock supplier facing many burdens from animal disease. This research investigates the economic effects of improving livestock health in Ethiopia. While many studies focus on a single disease [6–8], or diseases that affect a single species [9, 10], this work comprehensively considers mitigation of all diseases that affect cattle, sheep, and goats in Ethiopia to provide a necessary, comprehensive investigation of the burden of animal disease in the country [11]. We employ a computable general equilibrium model in tandem with animal health loss envelope (AHLE) estimates from the Global Burden of Animal Diseases (GBADs) compartmental livestock population model to simulate the economy-wide effects of two animal health improvement scenarios for cattle, sheep, and goat production in Ethiopia. This allows for investigating changes in Ethiopia’s domestic production, prices, trade, GDP, and economic welfare resulting from improved livestock health.

Animal health technology adoption and policy measures in response to animal disease events are designed to mitigate production losses and domestic and international market distortions, consequently affecting economic welfare. Many animal diseases are transboundary in nature and affect both developed and developing countries. Coordination and consistency in financing animal health measures are especially critical for transboundary diseases in developing countries [12]. There are noteworthy reasons why many developing countries are unlikely to make efforts toward eradicating animal diseases. Many developing countries do not have the capacity to export livestock and livestock products or are unable to prevent disease introduction from neighboring countries. Other developing economies import livestock and livestock products from countries with endemic animal diseases or predominantly extensive production systems, causing little incentive to eradicate animal diseases [13].

Animal disease control can be a crucial component of poverty-reduction strategies and represents a development opportunity in a globalized environment [14]. However, controlling animal diseases in smallholder systems in parts of Africa is challenging, particularly when movement restrictions are hard to enforce, and wildlife are endemically infected [15]. Small and medium-sized producers in developing countries tend to be disconnected from veterinary support systems [16]. The lack of infrastructure, human resources, and movement control in many developing countries creates environments that are particularly vulnerable to the spread of animal diseases [17, 18]. Accordingly, it is important to understand the economic effects of disease mitigation in developing countries. Ethiopia presents an important case study to investigate the implications of the burden of animal disease.

The remainder of this paper is organized as follows: the Literature review section presents the current state of knowledge regarding the economic effects of animal diseases, with a particular focus on developing countries. The Livestock production and animal diseases in Ethiopia section provides background information on Ethiopia’s livestock sector and the burden of animal diseases in the country. The Modeling framework and scenario design section describes the computable general equilibrium framework and the simulations applied in the model. The Results section presents the simulation results, including impacts on output, prices, trade, and macroeconomic outcomes. Finally, the last section concludes with policy implications and suggestions for future research.

## Literature review

Animal diseases have multifaceted impacts on food systems and economies, affecting food safety through product contamination, food security by reducing livestock productivity, and economic activity via trade restrictions and control costs. These complex effects require interdisciplinary research approaches, such as integrating epidemiological models with economic analysis to capture dynamic market effects [19]. However, accurately quantifying these impacts presents challenges due to disease-specific characteristics, data limitations (especially in developing countries), and indirect effects on market access and consumer behavior. It is crucial to consider both direct and indirect losses, as traditional cost-benefit analyses may underestimate total economic costs. Developing countries face additional challenges, including limited veterinary infrastructure, higher reliance on livestock for livelihoods, and constraints in implementing disease control measures. Consequently, there is a need for context-specific economic analyses in these countries, as models developed for industrialized agricultural systems may not be directly applicable [20, 21]. This complexity underscores the importance of comprehensive, tailored approaches to understanding and mitigating the economic impacts of animal diseases across diverse global contexts.

Perry et al. [22] identify three main trajectories in global trends in livestock diseases. The first trend consists of developed countries having well-controlled endemic diseases but increasing concerns about emerging threats. The second trend is rapidly developing countries experiencing intensification of livestock systems and complex disease patterns. The third trend shows less developed countries still struggling with endemic diseases in traditional smallholder systems. Key drivers of change include increasing demand for livestock products, urbanization, climate change, and globalization of trade. This calls for better integration of public and private sector efforts, improved disease surveillance, and consideration of environmental and social impacts alongside economic factors when formulating animal health policies.

Livestock play a crucial role in developing countries, contributing significantly to livelihoods, food security, and economic development. Nearly one billion people living on less than $2 per day in South Asia and sub-Saharan Africa keep livestock, with livestock contributing 33% of household income on average in mixed crop-livestock systems [23]. Livestock provide multiple benefits including income, food, traction, fertilizer, and savings. However, the sector also faces challenges related to environmental impacts, zoonotic disease risks, and production inefficiencies.

Globally, the economic burden of animal diseases is substantial. Estimates suggest that animal diseases cause annual production losses of around 20% worldwide, valued at approximately US$300 billion [24]. For instance, the 2001 FMD outbreak in the United Kingdom resulted in losses exceeding US$12 billion, with over half absorbed by non-agricultural sectors such as tourism [21]. In sub-Saharan Africa, where livestock contribute significantly to livelihoods, endemic diseases are estimated to cause annual losses of US$4 billion, or 25% of the total value of livestock production [25].

The economic impacts of animal diseases extend beyond direct production losses. They affect market access, especially for developing countries seeking to participate in international trade. For instance, the presence of FMD in most of sub-Saharan Africa, including Ethiopia, restricts most high-value export markets for beef [21]. This has significant implications for countries like Ethiopia, where livestock exports are an important source of foreign exchange [26].

Computable general equilibrium (CGE) models have become increasingly important tools for estimating the economy-wide impacts of animal diseases. These models capture the complex interactions between different sectors of the economy, providing a more comprehensive view of disease impacts than partial equilibrium models. For instance, Philippidis and Hubbard [27] use a CGE model to assess the impacts of the 2001 FMD outbreak in the United Kingdom, simulating a 0.8% decrease in gross domestic product (GDP), amounting to approximately £10 billion. De Menezes et al. [8] apply a CGE model to evaluate the economic impacts of potential FMD outbreaks in Brazil. Their study simulates GDP losses ranging from 0.02% to 0.09%, with particularly severe impacts on the livestock sector, where output could decrease by up to 3.92% in the most affected regions. In a related study, de Menezes et al. [28] project GDP losses between 0.08% and 0.32% resulting from FMD in Brazil, depending on the area affected by the disease, highlighting the regional variability of economic impacts.

Other applications of CGE models have provided valuable insights into disease impacts across different contexts. Wittwer [29] employs a CGE model to evaluate the economic impacts of a potential FMD outbreak in Australia, estimating that a large multi-state outbreak could result in a welfare loss between AUD$10 billion and AUD$85 billion depending on the duration of trade sanctions. Kompas et al. [30] apply a CGE model to assess the economy-wide impacts of African Swine Fever (ASF) in Vietnam and estimate that ASF could lead to a decrease in GDP of up to 0.7%. Oladosu et al. [31] use a CGE model to analyze several FMD outbreak scenarios in the U.S. Their results show that GDP losses could range from $37 billion to $228 billion, depending on outbreak severity and control measures [31]. Purcell et al. [32] apply a CGE model to Thailand’s economy, examining three scenarios: increased livestock production, increased exports, and improved productivity due to FMD control. They find that disease control programs would only yield significant economic benefits if coupled with the elimination of export restrictions [32].

The economic burden of animal diseases in developing countries, such as Ethiopia, is particularly severe and warrants careful study. In these nations, livestock often serve as a crucial lifeline for rural communities, contributing significantly to food security, income generation, and overall economic stability. Understanding the full economic impact of animal diseases in these contexts is essential for developing targeted, effective, and sustainable disease control strategies that can improve livelihoods and support economic development. In this regard, CGE models have proven valuable for understanding the broad economic implications of animal diseases. By capturing the intricate relationships between various economic sectors, these models offer critical insights that inform effective disease control strategies and policy decisions. The application of CGE models across different countries and contexts underscores their versatility and importance in understanding the economic impacts of animal diseases. For countries like Ethiopia, where resources are limited and the stakes are high, these models can help policymakers prioritize interventions, allocate resources more efficiently, and design policies that balance disease control with economic growth and poverty reduction.

## Livestock production and animal diseases in Ethiopia

Ethiopia has the second-largest human population in Africa and the largest livestock population on the continent. While Ethiopia is one of the fastest-growing African economies, approximately 24% of the population lives in extreme poverty [33]. About 80% of Ethiopian households depend on agriculture and have direct contact with livestock [34, 35]. Livestock production comprises 45% of the agricultural sector, 19% of the national GDP, and 20% of the country’s export earnings, making it indispensable for Ethiopia’s economy. At the rural household level, livestock is crucial for nutrition, food security, and livelihoods [36, 37]. Improvements in animal health that increase domestic livestock production directly benefit livestock-producing households and create indirect benefits throughout the supply chain and the entire economy.

**Fig 1:**
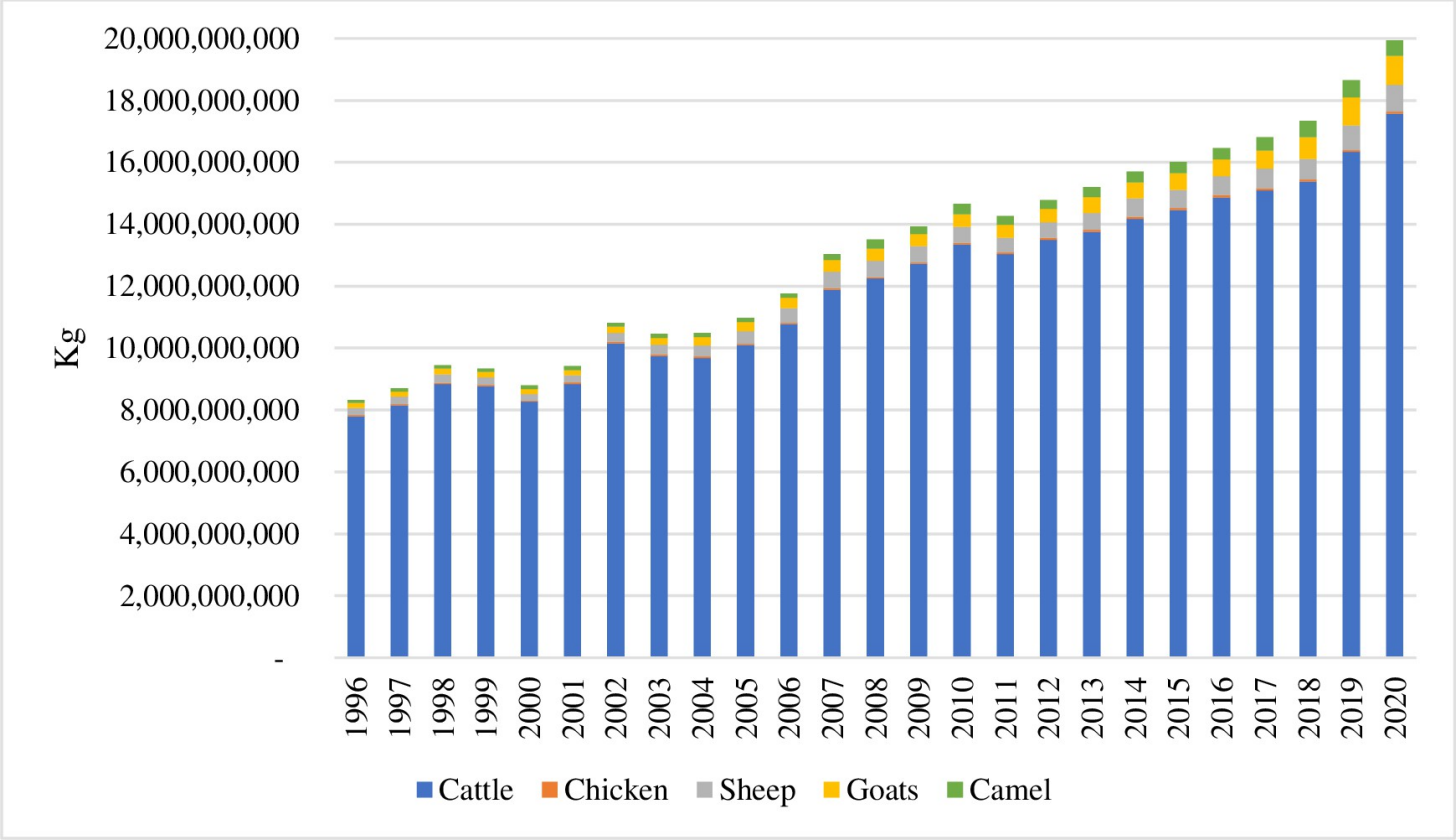
Ethiopian livestock biomass (Kg). Source: Authors’ calculations based on GBADs data [38].

Ethiopia’s livestock sector included approximately 70.3 million cattle, 57 million chickens, 52.5 million goats, 42.9 million sheep, and 1.6 million camels in 2020 [38]. This represents approximately 10% of Africa’s and 4% of the world’s small ruminant population [39]. Fig 1 highlights the distribution of Ethiopia’s livestock biomass among different species from 1996 to 2020. Currently, the two largest livestock sectors in Ethiopia are cattle and goats, followed by sheep and camels. Table 1 shows meat production in Ethiopia and Africa. Regarding production value, beef is the largest meat product, followed by goat, sheep, and chicken meat. Ethiopia’s gross production values for beef, goat, sheep, and chicken meat represent 8%, 24%, 6%, and 3% of Africa’s production, respectively.

**Table 1:** 2020 gross production value in Ethiopia and Africa (US$ 1,000).

Product	Ethiopia	Africa
Beef	980,672	12,218,178
Goat meat	400,471	1,652,819
Sheep meat	377,434	6,579,307
Chicken meat	282,429	10,731,197

Source: Authors’ calculations based on FAOSTAT data [40].

The contribution of livestock to foreign currency earnings is through exports of live animals, meat, skin, and hides [36]. Ethiopia’s live animal exports of cattle, sheep, and goats totaled $US 32.64 million in 2020. Beef, goat, and sheep meat exports totaled $64.83 million in 2020 [24]. Ethiopia’s meat production and export quantity are presented in Table 2. Beef, goat, and sheep meat exports represent 0.04%, 7.40% and 0.32% of production, respectively. Chicken meat is consumed domestically and not exported. Small ruminants make up about 25% of the value of meat produced in Ethiopia and provide substantial contributions to exports [39]. Despite Africa’s largest livestock population, Ethiopian production and exports are stifled. The development of the livestock sector is hampered by several constraints, including widespread endemic animal diseases that restrict trade. Therefore, the contribution of the livestock sector to household incomes and the national economy is well below potential [35].

**Table 2:** Ethiopia’s 2020 meat production and exports.

Product	Production (tons)	Exports	Exports
		(tons)	($US 1,000)
Beef	443,231.49	166.28	548.00
Goat meat	147,193.14	10,889.32	62,002.00
Sheep meat	131,724.90	418.81	2,283.00
Chicken meat	67,650.74	-	-

Source: Authors’ calculations based on FAOSTAT data [40].

Approximately 80% of farmers operate traditional mixed crop-livestock production systems in Ethiopia [41, 42]. Despite having a large population, small ruminant production in Ethiopia is largely based on traditional subsistence production with limited commercialization and modernization [39]. In addition, the scavenging chicken production system, which is the predominant production system in Ethiopia, is characterized by small flock size, minimal inputs, and low output. Disease-related mortality among livestock in Ethiopia is high and represents a major constraint to production [43]. Commercialization of livestock farming is still limited, though there are emerging trends towards more market-oriented production systems, especially in peri-urban areas and among larger-scale producers. This limited commercialization has important implications for our study, as it affects the adoption of disease control measures, access to veterinary services, and the overall economic impact of livestock diseases. Ethiopia is endemic to many livestock diseases, including diseases listed by the World Organization for Animal Health (WOAH), such as lumpy skin disease, FMD, bovine tuberculosis, Newcastle disease, pest des petits ruminants (PPR), and sheep and goat pox [44–47]. Jemberu et al. [6] estimate that FMD in Ethiopia leads to annual losses of US$1.35 billion in cattle production alone. In addition, Tschopp et al. [48] highlight the substantial economic burden of bovine tuberculosis in Ethiopia, which was estimated to cause an economic loss between $75.2 million and $358 million during 2005–2011, emphasizing the need for improved control strategies in the country.

Among the various livestock diseases affecting Ethiopia, PPR stands out as one of the most significant challenges, as highlighted in the 2015 Ethiopia Livestock Master Plan. This highly contagious viral disease primarily affects sheep and goats, with devastating consequences for small ruminant production. PPR is characterized by its severe impact, with morbidity and mortality rates reaching up to 90% in affected herds [49]. The economic burden of PPR on Ethiopian households is substantial, with reported losses equivalent to 14% of annual household income [39]. This underscores the critical need for effective disease control strategies, as PPR not only threatens livestock productivity but also significantly impacts the livelihoods of smallholder farmers who depend on small ruminants for their economic well-being.

In addition to affecting farming and livestock production, livestock diseases have other impacts on the highland mixed crop-livestock system, including decreased work performance of oxen, which contributes to severe food insecurity and poverty. Livestock diseases reduce income, affect livestock producers’ livelihoods, and jeopardize food security at local and national levels [10]. Despite substantial export demand for livestock and livestock products and Ethiopia’s potential to export, the prevalence of many trade-limiting transboundary livestock diseases limits access to the international market and makes Ethiopia vulnerable to disease-related trade bans [35, 50].

Despite having the largest livestock population in Africa, Ethiopia’s exports are relatively low in part due to the use of animals for draught power and due to various trade barriers [51]. Tariffs on livestock and meat products vary among Ethiopia’s trading partners, with some preferential agreements in place. For instance, under the Common Market for Eastern and Southern Africa (COMESA), Ethiopia enjoys reduced tariffs on livestock exports to member countries [10]. However, non-tariff measures (NTMs) pose a more significant challenge to Ethiopia’s livestock and meat trade. Strict sanitary and phytosanitary (SPS) requirements, particularly those related to foot-and-mouth disease and other transboundary animal diseases, often restrict access to lucrative markets in the Middle East and Europe [6]. The lack of a comprehensive animal identification and traceability system further complicates compliance with international standards [52]. Domestically, Ethiopia’s livestock marketing system is characterized by multiple layers of intermediaries and informal cross-border trade, which can hinder efficient market integration and compliance with formal trade regulations [53]. Furthermore, Ethiopia’s government implemented export taxes on leather products to encourage the leather manufacturing industry and improve the domestic value chain. However, export taxes create market distortions that limit export potential for Ethiopian livestock products [54]. These multifaceted trade policies and measures collectively shape Ethiopia’s participation in global livestock markets, often limiting the country’s ability to fully capitalize on its livestock resources.

There is a strong correlation between the burden of animal diseases and poverty when livestock is the primary source of household income, as is the case for many Ethiopians [37]. While there have been disease-specific studies to understand impacts on Ethiopian livestock sectors, this is the first study to our knowledge that provides a comprehensive analysis of the effects of widespread disease mitigation for cattle, sheep, and goats in Ethiopia. Accordingly, this research provides further understanding of the potential economic implications of mitigating the burden of animal diseases to highlight opportunities for the Ethiopian agricultural economy.

## Modeling framework and scenario design

This research employs a computable general equilibrium (CGE) model in tandem with results from the GBADs compartmental livestock population model calibrated for goats, sheep, and cattle in Ethiopia to simulate the economic effects of two disease mitigation scenarios. The standard Global Trade Analysis Project (GTAP) model and database [55, 56] are used for this analysis. The GTAP modeling framework has been used widely to investigate economic questions that are economy-wide, including the impacts of animal disease and sanitary trade barriers on the global economy [8, 57–59]. The advantages of using a CGE model are that there is an accounting of the intersectoral linkages among agents and sectors across regions throughout the global economy, the explicit treatment of bilateral trade flows, and detailed national-level welfare analysis for all regions in the model. While a partial equilibrium model allows greater sectoral detail [60–63], CGE models account for important linkages throughout the global economy [64]. This work focuses on the national effects of disease mitigation in Ethiopia as well as changes in international trade and global welfare, and the economy-wide nature of the GTAP model is appropriate for this research.

The GTAP model is a multi-region, multi-sector, general equilibrium framework representing economic activity for 141 countries/regions and 65 sectors. This framework uses a global Social Accounting Matrix (SAM) that integrates national SAMs for all countries/regions in the model. These SAMs provide a comprehensive, economy-wide data framework representing the economy of each country or region. Each national SAM in GTAP captures the circular flow of income and expenditure in the economy, being constructed through various national and international data sources and harmonized to create consistent SAMs across countries. One of the important advantages of using the GTAP framework in this context is that the model, data, and parameters are already specified for Ethiopia and other countries/regions, which overcomes noteworthy limitations for data availability in resource-constrained countries. Constraints imposed on the model ensure commodity and factor market clearing conditions hold and macroeconomic identities are maintained. The general equilibrium nature of the model allows for the investigation of the effects of exogenous shocks on endogenous variables, including equilibrium factor and commodity prices, changes in output, consumption, bilateral trade across sectors, and changes in national welfare across regions. Simulated shocks in any sector have repercussions throughout the economy because of interlinkages between sectors and agents, which is important for this work because changes in livestock health affect consumers and producers across the entire agricultural economy.

The GTAP production structure assumes perfect competition modeled as a sequence of nested Constant Elasticity of Substitution (CES) production functions, representing all substitution possibilities across the complete set of inputs [65]. Producers substitute primary factors as their relative prices change. Intermediate inputs enter the production function as a CES composite where intermediates from different regions are imperfect substitutes. Regional household demand is characterized by a Constant Difference of Elasticities specification, which allows income growth to affect consumer preferences [55, 66, 67]. Each region’s representative household maximizes utility derived from the consumption of market goods and savings subject to a regional income constraint. Cobb-Douglas functions describe government and investment demand. The model accounts for international trade and transport margins, where the bilateral differences between cost insurance and freight (*cif*) and free on board (*fob*) prices for each country are estimated using data from the U.S. Bureau of Census, Foreign Trade Statistics [56]. Bilateral trade is determined by the Armington import demand specification, where demand is first allocated between domestically produced goods and an import composite, followed by regional import sourcing of the composite import [68].

While single-country general equilibrium models provide detailed insights into a specific country’s economy, the GTAP model offers several advantages that make it particularly suitable for analyzing the economic impacts of animal diseases, especially in the context of a globally interconnected world. First, the GTAP model captures international bilateral trade flows and interdependencies between countries. This is crucial when studying animal diseases, as their impacts often extend beyond national borders through trade restrictions, price changes in global markets, and shifts in international demand. For a country like Ethiopia, which aims to expand its livestock exports, understanding these global linkages is essential. Second, GTAP allows for the analysis of spillover effects across countries and regions. An animal disease outbreak in one country can have significant economic consequences for its trading partners. The model can capture these indirect effects, providing a more comprehensive understanding of the global economic impacts of animal diseases. Third, GTAP provides a consistent and regularly updated global database, ensuring that the analysis is based on harmonized data across countries. This is especially valuable when investigating the economic effects of exogenous shocks in developing countries like Ethiopia, where comprehensive economic data might be limited or inconsistent. Finally, for policymakers in Ethiopia, understanding how their decisions fit into and are influenced by the global context is crucial. Employing the GTAP modeling framework provides this broader perspective, which is essential for developing robust, internationally aware policies.

Version 10 of the GTAP database includes 141 regions and 65 sectors of economic activity that can be described more generally by three broad categories: agriculture and food processing, manufacturing, and services. GTAP sectors are aggregated into 21 groups for this study by combining nonagricultural commodities into six categories, while 15 agricultural sectors are kept separate, as described in S1 Table. We assessed FAO and country-level profiles of Ethiopia to identify the most important sectors of the Ethiopian agricultural economy and determine how to aggregate the GTAP database for this work. We separated primary agriculture, fishing, forestry, and basic pharmaceutical sectors. The most important livestock animals and animal products in Ethiopia are represented in GTAP by two live animal sectors and two meat sectors. Cattle, sheep, and goats are included in one GTAP sector and the corresponding meat products in another, while the other animals and other animal product sectors primarily include pork and poultry. The other food and beverages sector includes processed agricultural, food, and beverage products in one combined sector. The remaining nonagricultural sectors include combined sectors for energy and extraction, manufacturing, and services.

The regional aggregation for the model is described in S2 Table. Countries in the GTAP database are aggregated into ten groups representing geographic regions of the world in addition to Ethiopia, which is modeled separately. This allows us to capture general trade patterns across regions of the world and prevents us from inadvertently focusing on Ethiopia’s individual trade partners. This work follows the standard approach of using macroeconomic shocks to update version 10 of the GTAP database from 2014 to 2021 by simulating observed changes in productivity and population growth [66, 67].

Two animal health improvement scenarios were designed based on animal health loss envelope (AHLE) estimates from the GBADs compartmental livestock population model for Ethiopia. Gilbert et al. [11] provide a methodological approach to the animal health loss envelope. Table 3 describes the share-weighted percentage changes in production for the combined GTAP sector for cattle, sheep, and goats in bold for each scenario. The table includes the share of total production for the sector in column two and the percentage change in the value of production for each scenario in columns three and four, which is used to calculate the weighted average change in the total value of production for the combined cattle, sheep, and goat GTAP sector. The Zero Mortality Scenario considers the direct changes in production for cattle, sheep, and goats if disease-related livestock mortality in Ethiopia were reduced to zero, where all other parameters remain the same as the baseline scenario for the livestock population model. The Ideal Scenario considers both direct and indirect impacts from animal health improvement to achieve zero mortality, and parameters in the compartmental livestock population model are adjusted for ideal health, resulting in larger changes in production. Given that the GTAP sector for this analysis includes multiple species, share-weighted averages using FAO production data were calculated to determine production shock values for the cattle, sheep, and goats GTAP sector. Production for the cattle, sheep, and goats GTAP sector is expected to increase by 180.48% for the Ideal Scenario and 40.20% for the Zero Mortality Scenario. The share-weighted production changes in bold are simulated in the GTAP model to understand the global economic impacts of two different scenarios for improved livestock health of cattle, sheep, and goats in Ethiopia.

**Table 3:** Share-weighted changes in production by GTAP sector by scenario.

		Percentage Change in Production	
	Sector Share	Ideal Scenario	Zero Mortality Scenario
Cattle	0.56	151.55%	13.38%
Goats	0.24	266.42%	97.10%
Sheep	0.19	156.06%	46.23%
**Cattle, Sheep, and Goats Sector**		**180.48%**	**40.20%**

Source: Authors’ calculations of share-weighted percentage changes in the cattle, sheep, and goats GTAP sector production in bold. Based on sector shares from FAOSTAT [40] and changes in production based on GBADs compartmental livestock population model for Ethiopia.

It is important to note that these scenarios simulate the potential benefits of improved animal health but do not account for the costs of interventions needed to achieve these improvements. This is a limitation of the current analysis, as implementing disease control measures would require significant investments in areas such as veterinary services, vaccination programs, and improved animal management practices. Future research is warranted to quantify these costs to allow for a comprehensive economic evaluation of potential animal health interventions in Ethiopia.

## Results

Simulated percentage changes in domestic output and market prices for the Ideal and Zero Mortality Scenarios are described in Table 4. We also provide S3 Table, which provides the corresponding absolute changes in the values of output, exports, and imports. As expected, the largest effects for both scenarios are for the cattle, sheep, and goat sector. However, there are relatively minor cross-sectoral impacts in general. We present the Ideal Scenario results, followed by the Zero Mortality Scenario results.

**Table 4:** Percentage changes in output and domestic market prices in Ethiopia.

Sector	Output		Domestic Market Prices	
	Ideal	Zero Mortality	Ideal	Zero Mortality
Paddy Rice	1.02	1.24	0.72	-0.31
Wheat	2.34	2.09	0.92	-0.24
Cereal Grains	2.19	1.49	0.94	-0.30
Vegetables, Fruit, Nuts	2.11	1.90	0.99	-0.29
Oilseeds	-2.05	1.60	0.58	-0.35
Sugar Cane and Beet	2.70	2.12	1.04	-0.24
Plant-based Fibers	2.49	2.40	0.96	-0.31
Other Crops	-0.72	1.84	0.71	-0.34
Cattle, Sheep, Goats	180.36	40.20	-80.56	-65.53
Other Animals	2.88	2.38	1.03	-0.28
Raw Milk	2.90	2.35	1.04	-0.27
Wool and Silk	1.76	2.14	0.89	-0.26
Meat: Cattle, Sheep, Goats	1.81	2.17	0.50	-0.22
Other Meat	1.94	1.82	1.37	0.40
Other Food and Beverages	2.81	2.20	1.56	0.56
Forestry	1.16	1.18	3.41	2.23
Fishing	2.05	1.55	8.64	5.96
Basic Pharmaceuticals	-0.72	0.70	1.03	0.29
Mining and Extraction	-0.64	0.04	1.20	0.87
Manufacturing	0.74	1.74	0.65	-0.08
Services	1.18	1.31	1.43	0.52

Source: Authors’ simulations

### Output and market prices

The Ideal Scenario leads to a simulated 180.36% increase in output of the cattle, sheep, and goat sector. The price change is also substantial, with the simulated price decrease equal to -80.56% for the cattle, sheep, and goat sector. Changes in output are relatively minor for other sectors. The simulated percentage changes in output for other agricultural sectors range from -2.05% for oilseeds to 2.90% for raw milk. Price changes for agricultural sectors other than the cattle, sheep, and goat sector are nominal, with simulated changes in prices of less than one percent. There is a simulated 2.81% increase in other food and beverage output in tandem with a 1.56% simulated price increase for the sector. The Ideal Scenario leads to simulated changes in output between -0.72% and 2.05% for nonagricultural sectors and larger price changes compared to agriculture. Changes in nonagricultural prices are approximately one percent except for forestry and fishing, with prices increasing by 3.41% and 8.64%, respectively.

Results for the Zero Mortality Scenario follow a similar pattern yet are smaller in magnitude than the Ideal Scenario, given the relatively smaller changes in production for the Zero Mortality Scenario determined by the disease simulation model. Simulated output for the cattle, sheep, and goat sector increases by 40.20%, along with a price decrease equal to -65.53%. Changes in output for other agricultural sectors are lowest for paddy rice (1.24%) and highest for plant-based fibers (2.40%). Price changes are negligible for other agricultural sectors and are negative except for a minor, less than one percent increase in the prices of other meat and other food and beverage. Other food and beverage output is simulated to increase by 2.20% with a corresponding 0.56% price increase. Forestry and fishing output are simulated to increase by 1.18% and 1.55%, respectively. Forestry and fishing have the largest non-livestock price increases equal to 2.23% and 5.96%, respectively. Changes in output for the remaining nonagricultural sectors are equal to 1.74% or less, with price changes equal to less than one percent.

The key results from both scenarios highlight that simulated changes in output and prices directly affect the live animal sectors with relatively small cross-sectoral impacts on national output. The increased output simulated for the cattle, sheep, and goat sector resulting from improved animal health causes noteworthy price changes that affect the livestock market. Increased production and decreased prices lead to substantial effects on cattle, sheep, and goat exports.

The different reactions of the cattle, sheep, and goat meat sector between the two scenarios highlight the complex, non-linear relationships captured by the CGE model. In the Ideal Scenario, the substantial increase in live cattle, sheep, and goat production (180.36%) leads to a significant drop in live animal prices (-80.56%), which substantially reduces input costs for meat production. However, the meat sector output increases by only 1.81%, with a slight price increase of 0.50%. This seemingly counterintuitive result can be explained by a few factors. First, it is important to recall the importance of the use of draught animals in Ethiopia, where increased live animal production provides additional draught capacity in addition to slaughter. Also, other constraints in cattle, sheep, and goat meat processing may limit the sector’s ability to expand output relative to the increase in live animal supply. The income effect from overall economic growth increases demand for meat, putting upward pressure on prices despite lower input costs. In contrast, the Zero Mortality Scenario shows a more modest increase in live cattle, sheep, and goat production (40.20%) and a smaller price decrease (-65.53%). In this case, output of the cattle, sheep, and goat meat sector increases by 2.17%, slightly more than in the Ideal Scenario, with a small price decrease of -0.22%. This scenario represents more balanced growth where the cattle, sheep, and goat meat sector can more readily absorb the increased animal supply. Similar non-linear effects can be observed in milk production, where the larger shock in the Ideal Scenario does not translate to proportionally larger increases in output compared to the Zero Mortality Scenario. The current scenario design only accounts for production improvements in live animal production and further research on additional supply chain impacts of improved animal health warrants future research.

It is important to note that while our model indicates significant potential for increased live animal production and lower prices, several factors could moderate these effects in practice. While there are endogenous changes throughout the economy in response to simulated livestock production changes, market reactions might include shifts in consumption patterns, with consumers potentially increasing their demand for animal products in response to changes in animal health or substituting towards other protein sources if the supply increase is perceived as temporary. Producers may adjust their herd management practices in response to changing market conditions, potentially tempering the production increase. Additionally, policy interventions aimed at stabilizing markets could mitigate extreme price fluctuations. It is also worth considering that improvements in animal health would likely occur gradually over time rather than simultaneously with a subsequent long-run adjustment throughout markets as modeled. Modeling assumptions and parameterization lead to limitations that merit consideration for continued research. Therefore, these results should be interpreted as indicative of the direction and relative magnitude of potential changes, rather than precise predictions.

### Exports and imports

Improved animal health in Ethiopia causes an increase in cattle, sheep, and goat exports and a decrease in livestock imports as illustrated in Table 5 and in S3 Table. However, it is important to note that while baseline exports are relatively low partly because of barriers to trade, we do not model export restrictions in our scenarios and exports increase endogenously in the model because the relative price for the Ethiopian cattle, sheep, and goat sector decreases substantially. Under the Ideal Scenario, imports of cattle, sheep, and goats decrease by -94.83%. Imports for other agricultural sectors increase from 1.88% for oilseeds to 7.37% for raw milk; the exception is a -0.82% decrease in imports of paddy rice. Imports of other food and beverages increase by 6.31%, and nonagricultural imports increase from 2.71% for basic pharmaceuticals to 13.49% for fishing for the Ideal Scenario. Exports of cattle, sheep, and goats increase substantially, by 2,476%, though it is important to put this large increase into perspective. Ethiopia is a relatively small exporter of live animals, and importantly, more than 90% of cattle, sheep and goat exports are destined for the Central and Southern Africa region. Results show that Ethiopian exports of cattle, sheep, and goats to Central and Southern Africa increase by 203% for the Ideal Scenario. Ethiopian exports of all the other agricultural products decrease, ranging from -2.35 for cereal grains to -11.22% for other meat for the Ideal Scenario. Nonagricultural exports also decrease, especially a noteworthy -18.69% decrease in fishing exports.

**Table 5:** Percentage changes in imports and exports in Ethiopia.

Sector	Imports		Exports	
	Ideal	Zero Mortality	Ideal	Zero Mortality
Paddy Rice	-0.82	-4.28	-6.81	2.56
Wheat	6.73	1.03	-7.66	1.99
Cereal Grains	4.19	1.80	-2.35	0.69
Vegetables, Fruit, Nuts	4.86	1.68	-2.51	0.63
Oilseeds	1.88	1.03	-2.63	1.54
Sugar Cane and Beet	5.73	1.53	-5.52	1.28
Plant-based Fibers	5.98	2.01	-4.70	1.53
Other Crops	5.32	1.06	-4.17	1.96
Cattle, Sheep, Goats	-94.83	-85.30	2,476.53	384.01
Other Animals	5.07	2.40	-2.40	0.64
Raw Milk	7.37	1.58	-7.31	1.97
Wool and Silk	8.26	0.58	-10.89	3.33
Meat: Cattle, Sheep, Goats	3.78	1.13	-3.85	1.53
Other Meat	7.27	3.24	-11.22	-3.41
Other Food and Beverages	6.31	3.46	-6.31	-2.34
Forestry	10.71	7.04	-15.13	-10.24
Fishing	13.49	9.27	-18.69	-13.45
Basic Pharmaceuticals	2.71	1.71	-6.50	-1.91
Mining and Extraction	6.75	5.70	-12.84	-9.51
Manufacturing	3.07	1.43	-4.47	0.57
Services	4.00	2.30	-5.09	-1.90

Source: Authors’ simulations

The Zero Mortality Scenario leads to more muted changes in agricultural imports and exports relative to the Ideal Scenario. Cattle, sheep, and goat imports decrease, as expected, with a decline equal to -85.30%. Imports increase for all other agricultural products except for a -4.28% decline in paddy rice imports. Imports for other agricultural sectors increase ranging from 0.58% for wool and silk to 3.46% for other food and beverage imports. Imports increase for all nonagricultural sectors. Forestry and fishing imports increase the most and rise by 7.04% and 9.27%, respectively. Imports of basic pharmaceuticals, mining and extraction, manufacturing, and services increase by 1.71%, 5.70%, 1.43%, and 2.30%, respectively.

Total exports of cattle, sheep, and goats increase by 384.01% for the Zero Mortality Scenario. Again, the most important export destination is the Central and Southern Africa region, which comprises more than 90% of Ethiopia’s cattle, sheep, and goat exports. Exports to the Central and Southern Africa region are simulated to increase by 141% for the Zero Mortality Scenario. Other agricultural product exports increase, except for other meat and other food and beverages, which decrease by -3.41% and -2.34%, respectively under the Zero Mortality Scenario. Exports decrease for all nonagricultural products, except manufacturing, which has a minor 0.57% export increase. Exports of forestry, fishing, and mining and extraction decrease by -10.24%, -13.45%, and -9.51%, respectively. Exports also fall for basic pharmaceuticals (-1.91%) and services (-1.90%).

The increase in imports of animal products observed in our scenarios of increased animal productivity may seem counterintuitive at first glance. However, this result can be explained by factors within the model’s framework. First, the improved productivity in the livestock sector leads to overall economic growth, resulting in an income effect. This increase in national income can drive up demand for all goods, including imported animal products, as consumers’ purchasing power improves. Second, the GTAP model employs the Armington assumption, which treats domestic and imported goods as imperfect substitutes. This means that even with increased domestic production, there may still be increased demand for imported varieties given consumer preferences and changes in relative prices. As previously noted, model and parameter assumptions lead to limitations in the economy-wide framework.

### Macroeconomic outcomes and economic welfare

The percentage changes in GDP and the absolute change in regional welfare measured by equivalent variation across regions in the model are included in Table 6. GDP measures the monetary value of final goods and services produced in a country for a given period, and equivalent variation is a money-metric measure of economic welfare that describes the additional level of income needed for a region to be equally as well off at current income and price levels as would be the case after a change in the economy that affects income and prices. The decomposition of welfare in Table 7 describes changes attributable to allocative efficiency gains associated with the reallocation of resources in response to scenarios for improved livestock health, technological change, terms of trade, and changes in savings and investment. Both animal health improvement scenarios benefit the Ethiopian economy in terms of GDP and welfare. The Ideal Scenario leads to simulated gains in GDP equal to 3.57%, which is equivalent to $3.97 billion when considering Ethiopia’s GDP in 2021. The Zero Mortality Scenario results in a 2.51% GDP gain, which is equal to $2.79 billion for Ethiopia. Welfare gains for the nation are equal to more than $2.4 billion for the Ideal Scenario and $1.7 billion for the Zero Mortality Scenario. In other words, the increase in Ethiopian welfare ranges between 1.53% and 2.16% of Ethiopia’s GDP in 2021.

**Table 6:** Changes in GDP and welfare measured by equivalent variation.

Region	GDP (%)		Welfare ($US million)	
	Ideal	Zero Mortality	Ideal	Zero Mortality
Ethiopia	3.57	2.51	2,452.18	1,760.05
Former Soviet Union	0.00	0.00	30.22	10.00
Rest of Europe	0.00	0.00	134.61	46.85
Middle East and North Africa	0.00	0.00	62.71	37.75
Central and Southern Africa	0.00	0.00	230.91	170.03
China and Hong Kong	0.00	0.00	53.27	28.20
Southeast Asia	0.00	0.00	20.70	7.66
South Asia	0.00	0.00	-5.69	-2.64
Rest of Asia and Oceania	0.00	0.00	9.35	6.57
North America	0.00	0.00	-22.40	-20.35
Central and South America	0.00	0.00	0.58	-5.40

Source: Authors’ simulations

Note: Equivalent variation is a money-metric measure of welfare that describes the additional level of income needed for a region to be equally as well off at current income and price levels and would be the case after a change in the economy that affects income and prices.

**Table 7:** Decomposition of welfare changes (Equivalent Variation, $US Million).

Region	Allocative Efficiency		Technological Change		Terms of Trade		Changes in Savings and Investment		Total	
	Ideal	Zero Mortality	Ideal	Zero Mortality	Ideal	Zero Mortality	Ideal	Zero Mortality	Ideal	Zero Mortality
Ethiopia	185.49	107.37	2,632.28	1,874.26	-391.01	-206.75	25.42	-14.83	2,452.18	1,760.05
Former Soviet Union	7.51	1.81	0.00	0.00	27.84	10.53	-5.13	-2.34	30.22	10.00
Rest of Europe	66.18	19.43	0.00	0.00	78.54	30.42	-10.11	-3.00	134.61	46.85
Middle East and North Africa	5.31	1.63	0.00	0.00	68.24	39.40	-10.84	-3.28	62.71	37.75
Central and Southern Africa	36.48	26.06	0.00	0.00	189.51	139.53	4.92	4.44	230.91	170.03
China and Hong Kong	20.55	5.54	0.00	0.00	39.80	14.40	-7.08	8.26	53.27	28.20
Southeast Asia	-0.74	-0.1	0.00	0.00	22.00	6.97	-0.56	0.79	20.7	7.66
South Asia	2.48	1.38	0.00	0.00	-12.85	-7.08	4.68	3.06	-5.69	-2.64
Rest of Asia and Oceania	3.24	1.61	0.00	0.00	13.60	7.42	-7.49	-2.46	9.35	6.57
North America	10.77	1.7	0.00	0.00	-41.28	-30.97	8.11	8.92	-22.4	-20.35
Central and South America	0.75	0.03	0.00	0.00	1.18	-5.76	-1.35	0.33	0.58	-5.40

Source: Authors’ simulations

The welfare gains for Ethiopia are substantial because of the technological efficiency gains resulting from improved livestock health in the country. Both animal health improvement scenarios were modeled as changes in technology associated with livestock production. Accordingly, welfare gains predominately occur due to simulated technological improvement and contribute more than $2.6 billion and nearly $1.9 billion to national welfare for the Ideal and Zero Mortality Scenarios, respectively. There are also relatively small welfare gains related to allocative efficiency, where Ethiopia’s resources are redistributed to more efficient uses after the technology improvement in the cattle, sheep, and goat sector. The Ideal Scenario generates $185 million in welfare gains because of allocative efficiency improvements while allocative efficiency given the Zero Mortality Scenario contributes $107 million to welfare in Ethiopia.

Terms of trade measures the price of a region’s exports relative to the price of imports. The negative terms of trade effects for Ethiopia, which are equal to -$391 million and -$207 million for the Ideal and Zero Mortality Scenarios, respectively, indicate a redistribution of global import purchasing capabilities at the expense of Ethiopia. The final category for contributions to regional welfare is the change in the price of domestic capital investment goods relative to the price of savings. In Ethiopia, there are very minor changes with a positive welfare contribution from changes in savings and investment equal to $25 million for the Ideal Scenario, meaning that there was an increase in the price of domestic capital relative to the price of savings. However, the Zero Mortality Scenario reveals the opposite, with a small but negative welfare contribution of approximately -$15 million from changes in savings and investment for the Zero Mortality Scenario.

Improved animal health in Ethiopia has minor GDP and welfare effects across other regions. The Middle East and North Africa, Central and Southern Africa, and Rest of Europe experience the largest welfare gains, ranging from 0.0010% to 0.013% of their respective 2021 GDPs across scenarios. China and Hong Kong see modest gains, while other regions like the Former Soviet Union, Rest of Asia and Oceania, Southeast Asia, and Central and South America experience smaller positive effects. North America and South Asia face slight negative welfare impacts. The magnitude of these effects is generally larger in the Ideal Scenario compared to the Zero Mortality Scenario. The gains and losses in welfare for all regions outside Ethiopia are primarily a result of improved, or worsened terms of trade, as well as minor contributions from allocative efficiency and changes in savings and investment. In general, the world would have a total welfare gain of nearly $3 billion in the Ideal Scenario and more than $2 billion in the Zero Mortality Scenario.

It is noteworthy that since the GTAP model uses equivalent variation as its welfare measure, welfare changes are an economy-wide metric. This measure represents the overall welfare change for the entire economy, not distinguishing between different economic agents. In addition, the model calculates net welfare changes for a single representative household for each region, which combines consumers, producers, and the government into one entity for welfare calculations. GTAP captures complex interactions across all sectors simultaneously, therefore, welfare changes result from these interconnected effects, making it difficult to isolate impacts on specific groups or sectors. These features allow GTAP to provide powerful insights into overall economic impacts but limit its ability to disaggregate welfare effects among different economic agents or sectors.

Our findings for the potential economic benefits of improving animal health in Ethiopia align with and extend previous research on this topic in developing countries. For instance, our projected GDP gains of 2.5% to 3.6% are comparable to, albeit higher than, those found by Kompas et al. [30] for Vietnam, where they estimated that eradicating foot-and-mouth disease could lead to a GDP increase of up to 0.7%. The larger impact in our study likely reflects the greater importance of livestock for Ethiopia’s economy compared to Vietnam’s and the fact that we consider mitigation of all animal diseases rather than one specific disease as is the case in the Kompas et al. [30] study. Our results also echo the findings of Jemberu et al. [6], who estimated that FMD alone causes annual losses of US$1.35 billion in Ethiopian cattle production. Our more comprehensive approach, considering all diseases affecting cattle, sheep, and goats, naturally yields larger potential benefits when considering animal disease mitigation in Ethiopia. The substantial export gain we project for the cattle, sheep and goat sector (384% for the Zero Mortality Scenario) is larger than the 28.18% increase in livestock exports from Thailand resulting from FMD control found by Purcell et al. [32]. Our larger estimates again reflect both the broader scope of diseases considered and the critical role of livestock in Ethiopia’s economy. Furthermore, our findings for potential export increases align with Rich and Perry’s [21] assertion that improved animal health can significantly enhance developing country participation in international livestock markets. The authors note that international markets for beef are segmented based on FMD status, with higher price premiums available for countries that are FMD-free and do not vaccinate. They state that the presence of endemic FMD in most of sub-Saharan Africa closes off access to high-value export markets like the EU, the U.S., Japan, and Korea. Rich and Perry [21] also highlight that animal diseases reduce productivity through mortality, morbidity, and production losses. Therefore, according to the authors, controlling diseases allows for increased production and export through the establishment of trust and credibility as a reliable trading partner. Our study, however, goes beyond most previous research by providing a more comprehensive, economy-wide assessment of animal health improvements, capturing not only direct effects on the livestock sector but also spillover effects on other sectors of the Ethiopian economy.

## Conclusions and policy implications

Animal diseases cause decreased productivity and economic losses worldwide and are especially detrimental to developing economies that depend on livestock to sustain livelihoods and meet food security needs. Developing countries lack the resources for animal disease surveillance and mitigation, resulting in endemic disease presence that negatively impacts production, international market opportunities, and income. There are economic gains to be achieved through disease mitigation, and this study provides insight into the economy-wide impacts of animal health improvement in a developing country context by simulating livestock disease mitigation scenarios for cattle, sheep, and goats in Ethiopia.

The livestock sector is central to the Ethiopian economy and livelihoods, with Ethiopia hosting the largest livestock herd in Africa. However, animal diseases significantly constrain production and economic benefits, linking disease burden to poverty [51, 69–71]. Endemic diseases hinder exports despite international demand [72], and direct economic losses from cattle diseases are substantial [73]. Compounding these issues, livestock producers face limited access to animal health services, with less than 50% of cattle producers in some regions having access to veterinary services and regular vaccination programs [74]. While previous studies have estimated disease-related losses, further research on the economy-wide effects of mitigating animal disease burdens in Ethiopia is warranted. This paper addresses this gap by examining the comprehensive economic impacts of livestock disease burden in Ethiopia. To understand the economic effects of improved livestock health, this research simulates two scenarios determined by a disease simulation model in a computable general equilibrium modeling framework. The Ideal Scenario considers both the direct and indirect effects of health improvement in the livestock supply chain, while the Zero Mortality Scenario considers only the direct effects of eliminating disease-related mortality for cattle, sheep, and goats.

Results show that livestock disease mitigation can potentially increase cattle, sheep, and goat production by 40%-180%, at the expense of depressed domestic prices ranging from -65% to -80% compared to baseline prices. Improved animal health can lead to oversupply when livestock inventories remain unchanged, which should be considered in planning scenarios and policy implementation. As countries adapt to improved health, a historical trend has been to realize increased production per head and decreased herd inventories, which helps to mitigate oversupply problems. Increased output also leads to noteworthy export growth for cattle, sheep, and goats. Ethiopian GDP has the potential to increase by 2.5% to 3.6% across scenarios. This comes in tandem with welfare gains ranging from more than $1.7 billion to nearly $2.5 billion for Ethiopia. The substantial GDP and welfare gains simulated by both scenarios result from the importance of livestock in Ethiopia, which comprises 19% of the national economy. Other regions are also expected to benefit from improved animal health in Ethiopia, especially Central and Southern Africa, with an increase in welfare between $170 million and $231 million.

This study’s findings have several important policy implications that center on the potential for improved animal health systems in Ethiopia. First, results underscore the importance of investment in animal health services, including considerations for expanding access to veterinary care and vaccination programs, particularly in rural areas, which would be necessary to achieve the simulated animal health improvements across the country. Second, this work highlights the potential value of implementing other mechanisms to achieve animal health improvements which could include a comprehensive national animal health strategy that integrates disease surveillance, biosecurity measures, and farmer education programs, for example. Third, our results suggest that improvements in animal health could have significant positive spillover effects on other sectors of the economy, indicating that such investments could be an effective lever for broader economic development.

While this research provides an initial contribution to understanding the broader economic effects of animal disease mitigation in a developing country context, further investigation is warranted. First, we assume improvements in the Ethiopian livestock sector but not in other countries. Second, the scenarios considered in this analysis account for productivity changes in the cattle, sheep, and goat GTAP sector, and there are additional changes along the livestock and meat supply chain that merit consideration in the scenario design for future work. Third, this study does not account for the costs associated with livestock health improvement or additional benefits that are not considered in this work, including potential positive human health impacts associated with zoonotic diseases. Unlike human health, government expenditure on animal health is not reported systematically across countries, hindering optimal resource allocation. This is an important limitation of the current study. Realizing the benefits simulated in our scenarios would likely require significant investments and policy measures. For example, expanding access to veterinary services, particularly in rural areas, implementing comprehensive vaccination programs for major livestock diseases, improving animal identification and traceability systems, enhancing biosecurity measures at the farm level; strengthening disease surveillance and reporting systems, investing in education and training for livestock producers on disease prevention and management, and developing policies to incentivize adoption of improved animal health practices are potential mechanisms to achieve improved animal health in Ethiopia. Future research is needed to quantify the costs associated with potential interventions and associated benefits of various measures to expand the analyses of the national effects associated with livestock disease mitigation in Ethiopia and other countries. This would provide policymakers with more complete information for decision-making regarding investments in animal health improvement programs. Additionally, achieving the levels of animal health improvement simulated in our scenarios would likely require a phased approach over several years. Future studies could also explore the potential economic impacts of gradual improvements in animal health, accounting for the time and resources needed to implement interventions effectively. National policy to facilitate improved livestock health or animal-health-related producer support programs may provide opportunities to improve the livelihoods of livestock producers in tandem with improved national food security. Continued study is warranted to understand the complex economic implications of animal disease in Ethiopia and other developing economies.

## Supporting information

S1 Table GTAP sectoral aggregation. (PDF)

S2 Table GTAP regional aggregation. (PDF)

S3 Table Value changes for Ethiopia’s Output, exports, and imports (in $1,000). (PDF)

S1 Dataset (ZIP)
