# Supplementary material for: Economic effects of livestock disease burden in Ethiopia: A computable general equilibrium analysis
Source: PLoS One. 2024 Dec 31;19(12):e0310268. doi: 10.1371/journal.pone.0310268 (PMC11687651; doi:10.1371/journal.pone.0310268)
Supplement: S1 Table — (PDF) [file pone.0310268.s001.pdf]

S1 Table. GTAP Sectoral Aggregation

Sectors Modeled	GTAP Sectors
Paddy Rice	Paddy rice
Wheat	Wheat
Cereal Grains	Cereal grains nec
Vegetables, Fruit, Nuts	Vegetables, fruit, nuts
Oilseeds	Oil seeds
Sugar Cane and Beet	Sugar cane, sugar beet
Plant-based Fibers	Plant-based fibers
Other Crops	Crops nec
Cattle, Sheep, Goats	Bovine cattle, sheep, and goats
Other Animals	Animal products nec
Raw Milk	Raw milk
Wool and Silk	Wool, silk-worm cocoons
Meat: Cattle, Sheep, Goats	Bovine meat products
Other Meat	Meat products nec
Other Food and Beverages	Vegetable oils and fats, Dairy products, Processed rice, Sugar, Food products nec, Beverages and tobacco products
Forestry	Forestry
Fishing	Fishing
Mining and Extraction	Coal, Oil, Gas, Minerals nec
Basic Pharmaceuticals	Basic pharmaceutical products
Manufacturing	Textiles, Wearinig apparel, Leather products, Wood products, Paper products, publishing, Petroleum, coal products, Mineral products nec, Ferrous metals, Metals nec, Metal products, Computer, electronic and optic, Electrical equipment, Machinery and equipment nec, Motor vehicles and parts, Transport equipment nec, Manufactures nec
Services	Electricity, Gas manufacture, distribution, Water, Contruction, Trade, Accommodation, Food and servic, Transport nec, Water transport, Air transport, Warehousing and support activities, Communication, Financial services nec, Insurance, Real estate activities, Business services nec, Recreational and other services, Public Administration and defense, Education, Human health and social work activities, Dwellings
