# Supplementary material for: Economic effects of livestock disease burden in Ethiopia: A computable general equilibrium analysis
Source: PLoS One. 2024 Dec 31;19(12):e0310268. doi: 10.1371/journal.pone.0310268 (PMC11687651; doi:10.1371/journal.pone.0310268)
Supplement: S2 Table — (PDF) [file pone.0310268.s002.pdf]

S2 Table. GTAP Regional Aggregation

Aggregation	Countries/Regions
Ethiopia	Ethiopia
Former Soviet Union	Belarus, Russian Federation, Ukraine, Rest of Eastern Europe, Kazakhstan, Kyrgyzstan, Tajikistan, Armenia, Azerbaijan, Georgia, Rest of Former Soviet Union
Rest of Europe	Austria, Belgium, Bulgaria, Croatia, Cyprus, Czech Republic, Denmark, Estonia, Finland, France, Germany, Greece, Hungary, Ireland, Italy, Latvia, Lithuania, Luxembourg, Malta, Netherlands, Poland, Portugal, Romania, Slovakia, Slovenia, Spain, Sweden, United Kingdom, Switzerland, Norway, Albania, Rest of EFTA, Rest of Europe
Middle East and North Africa	Bahrain, Iran Islamic Republic, Israel, Jordan, Kwait, Oman, Qatar, Saudi Arabia, Turkey, United Arab Emirates, Rest of Western Asia, Egypt, Morocco, Tunisia, Rest of North Africa
Central and Southern Africa	Benin, Burkina Faso, Cameroon, Cote d'Ivoire, Ghana, Guinea, Nigeria, Senegal, Togo, Rest of Western Africa, Central Africa, South Central Africa, Kenya, Madagascar, Malawi, Mauritius, Mozambique, Rwanda, Tanzania, Uganda, Zambia, Zimbabwe, Rest of Eastern Africa, Botswana, Namibia, South Africa, Rest of South Africa, Rest of the World
China and Hong Kong	China, Hong Kong
Southeast Asia	Cambodia, Indonesia, Lao People's Democratic Republic, Malaysia, Philippines, Singapore, Thailand, Viet Nam, Rest of Southeast Asia
South Asia	Bangladesh, India, Nepal, Pakistan, Sri Lanka, Rest of South Asia
Rest of Asia and Oceania	Australia, New Zealand, Rest of Oceania, Japan, Korea, Mongolia, Taiwan, Brunei Darussal, Rest of East Asia
North America	Canada, United States of America, Mexico, Rest of North America
Central and South America	Argentina, Bolivia, Brazil, Chile, Colombia, Ecuador, Paraguay, Peru, Uruguay, Venezuela, Rest of South America, Costa Rica, Guatemala, Honduras, Nicaragua, Panama, El Salvador, Rest of Central America, Dominican Republic, Jamaica, Puerto Rico, Trinidad and Tobago, Caribbean
