# Supplementary material for: Economic effects of livestock disease burden in Ethiopia: A computable general equilibrium analysis
Source: PLoS One. 2024 Dec 31;19(12):e0310268. doi: 10.1371/journal.pone.0310268 (PMC11687651; doi:10.1371/journal.pone.0310268)
Supplement: S3 Table — (PDF) [file pone.0310268.s003.pdf]

## S3 Table. Value Changes for Ethiopia’s Output, Exports, and Imports (in $1,000)

| Sector | Output | | Exports | | Imports | |
|---|---|---|---|---|---|---|
| | Ideal | Zero Mortality | Ideal | Zero Mortality | Ideal | Zero Mortality |
| Paddy Rice | 31.3 | 38.1 | -0.2 | 0.1 | 0.0 | -0.1 |
| Wheat | 37,814.4 | 33,774.4 | -1,041.8 | 270.6 | 955.7 | 146.3 |
| Cereal Grains | 147,299.4 | 100,217.4 | -907.1 | 266.3 | 1,697.0 | 729.0 |
| Vegetables, Fruit, Nuts | 158,503.2 | 142,728.0 | -26,329.9 | 6,608.7 | 67,262.4 | 23,251.2 |
| Oilseeds | -11,193.0 | 8,736.0 | -30,771.0 | 18,018.0 | 27,391.6 | 15,007.1 |
| Sugar Cane and Beet | 7,479.0 | 5,872.4 | -28.8 | 6.7 | 30.3 | 8.1 |
| Plant-based Fibers | 10,582.5 | 10,200.0 | -257.1 | 83.7 | 329.5 | 110.8 |
| Other Crops | -15,955.2 | 40,774.4 | -69,597.3 | 32,712.4 | 97,781.6 | 19,482.8 |
| Cattle, Sheep, Goats | 4,463,910.0 | 994,950.0 | 4,061,509.2 | 629,776.4 | -330,956.7 | -297,697.0 |
| Other Animals | 42,537.6 | 35,152.6 | -1,735.2 | 462.7 | 4,050.9 | 1,917.6 |
| Raw Milk | 41,325.0 | 33,487.5 | -296.1 | 79.8 | 298.5 | 64.0 |
| Wool and Silk | 11,369.6 | 13,824.4 | -482.4 | 147.5 | 371.7 | 26.1 |
| Meat: Cattle, Sheep, Goats | 11,077.2 | 13,280.4 | -4,735.5 | 1,881.9 | 4,951.8 | 1,480.3 |
| Other Meat | 4,151.6 | 3,894.8 | -341.1 | -103.7 | 224.6 | 100.1 |
| Other Food and Beverages | 208,726.8 | 163,416.0 | -5,622.2 | -2,084.9 | 5,944.0 | 3,259.3 |
| Forestry | 43,500.0 | 44,250.0 | -6,581.6 | -4,454.4 | 4,851.6 | 3,189.1 |
| Fishing | 40,077.5 | 30,302.5 | -596.2 | -429.1 | 430.3 | 295.7 |
| Basic Pharmaceuticals | -1,944.0 | 1,890.0 | -379.0 | -111.4 | 159.3 | 100.5 |
| Mining and Extraction | -6,476.8 | 404.8 | -10,220.6 | -7,570.0 | 6,081.8 | 5,135.7 |
| Manufacturing | 161,186.8 | 379,006.8 | -65,843.1 | 8,396.1 | 47,216.6 | 21,993.4 |
| Services | 891,726.0 | 989,967.0 | -175,859.5 | -65,645.0 | 138,200.0 | 79,465.0 |

Source: Authors’ simulations

Note: Changes in value correspond to model-simulated percentage changes in the value of output, value of exports, and value of imports for the Ideal and Zero Mortality Scenarios.
